# Legal assessment tool (LAT): an interactive tool to address privacy and data protection issues for data sharing

**DOI:** 10.1186/s12911-016-0325-0

**Published:** 2016-07-07

**Authors:** Wolfgang Kuchinke, Christian Krauth, René Bergmann, Töresin Karakoyun, Astrid Woollard, Irene Schluender, Benjamin Braasch, Martin Eckert, Christian Ohmann

**Affiliations:** 1 Heinrich-Heine-University, Düsseldorf, Germany; 2 TMF e.V, Berlin, Germany; 3 Isis Innovation Ltd, Oxford, UK; 4 ECRIN, KKS, Düsseldorf, Germany

## Abstract

**Background:**

In an unprecedented rate data in the life sciences is generated and stored in many different databases. An ever increasing part of this data is human health data and therefore falls under data protected by legal regulations. As part of the BioMedBridges project, which created infrastructures that connect more than 10 ESFRI research infrastructures (RI), the legal and ethical prerequisites of data sharing were examined employing a novel and pragmatic approach.

**Methods:**

We employed concepts from computer science to create legal requirement clusters that enable legal interoperability between databases for the areas of data protection, data security, Intellectual Property (IP) and security of biosample data. We analysed and extracted access rules and constraints from all data providers (databases) involved in the building of data bridges covering many of Europe’s most important databases. These requirement clusters were applied to five usage scenarios representing the data flow in different data bridges: Image bridge, Phenotype data bridge, Personalised medicine data bridge, Structural data bridge, and Biosample data bridge. A matrix was built to relate the important concepts from data protection regulations (e.g. pseudonymisation, identifyability, access control, consent management) with the results of the requirement clusters. An interactive user interface for querying the matrix for requirements necessary for compliant data sharing was created.

**Results:**

To guide researchers without the need for legal expert knowledge through legal requirements, an interactive tool, the Legal Assessment Tool (LAT), was developed. LAT provides researchers interactively with a selection process to characterise the involved types of data and databases and provides suitable requirements and recommendations for concrete data access and sharing situations. The results provided by LAT are based on an analysis of the data access and sharing conditions for different kinds of data of major databases in Europe.

**Conclusions:**

Data sharing for research purposes must be opened for human health data and LAT is one of the means to achieve this aim. In summary, LAT provides requirements in an interactive way for compliant data access and sharing with appropriate safeguards, restrictions and responsibilities by introducing a culture of responsibility and data governance when dealing with human data.

**Electronic supplementary material:**

The online version of this article (doi:10.1186/s12911-016-0325-0) contains supplementary material, which is available to authorized users.

## Background

### Medical research data makes up an increasing part of data sharing in the life sciences

In the life sciences an increasing part of stored data is medical data derived from humans. Researchers must effectively access and link this expanding volume of human data stored in various databases, repositories, and patient registries, in ways that make them available for analysis while protecting privacy and the legitimate interests of patients as well as data providers. We distinguish between data protection (privacy and confidentiality) and data security (protection against attacks, damage and unauthorised access). In our model for the process of data sharing as part of data bridges we consider two main roles: data provider and data consumer. The data provider is a database that offers services like data access and download of data. In case of the involvement of personal and sensitive data, the data provider becomes a data controller conformable to the European Data Protection Directive [1]. The researcher who used the data provided by the database for a well-defined research purpose is the data consumer. When the researcher builds-up an own database and gives access to his data he becomes a data provider. Several approaches for protecting human data have been suggested ranging from the creation of a political framework in the form of resolutions or treaties, and operational guidelines for data sharing in public health [2], to proposals for national privacy protection frameworks. These frameworks include concepts like legitimate public health purpose, minimum information necessary, privacy and security standards, data use agreements [3], ethical codes like the IMIA (International Medical Informatics Association) Code of Ethics for Health Information Professionals [4] and AMIA’s (American Medical Informatics Association) Code of Professional and Ethical Conduct [5], special guidance for genetic / genomic data covering recent controversies around their complete de-identifiability, and potential privacy risks originating from unintended uses [6]. Additional approaches exist, like the human rights-based approach to an international code of conduct for genomic and clinical data sharing [7], recommendations how open clinical databases can be adopted to strengthen privacy protections [8], and the iDASH (integrating Data for Analysis, Anonymization, and Sharing) Healthcare Privacy Protection Challenge that apply differentially-private methods [9, 10].

### Building data bridges between research infrastructures (RIs)

BioMedBridges [11] is a project that connects more than 10 ESFRI (European Strategy Forum on Research Infrastructures) research infrastructures (RIs) (e.g. ELIXIR, BBMRI (Biobanking and BioMolecular resources Research Infrastructure), EATRIS (European Advanced Translational Research Infrastructure in Medicine), ECRIN (European Clinical Research Infrastructures Network), Infrafrontier (Mouse Disease Models)) [12] to create so-called data bridges enabling data exchange and collaborative data sharing. Although, most data exchange in RIs consists of sharing animal data, collaboration between RIs is increasingly based on the integration of heterogeneous data sources that contain human health data. Thus the researcher is increasingly confronted with the above mentioned challenges of data and privacy protection and the understanding of the corresponding legal concepts. To examine the situation of data sharing of human health data for all BioMedBridges member research infrastructures, a survey was conducted at the beginning of the project [13]. We distinguished between the roles of data producer (an individual or group that generates data and uploads the data to a database), data provider (the database that stores the data and provides access to its data) and the data consumer (an individual or group that receives data in the form of a collection to use it for query, analysis, and reporting) [14]. The answers showed that about 36 % of the research institutions that constitute BioMedBridges members provided human data in their databases or imported human data and that about 79 % of the members are data consumers using human data or metadata for their research activities. This result indicates that there seems to be a trend in biomedical research towards an increased use of human data and therefore, it is safe to expect that more and more databases will include human data and that for research more and more human health data will be exchanged in future [15].

### Need for open access and legally interoperable data sharing

RIs are complex, international infrastructures consisting of member institutions maintaining databases and acting as data providers and data consumers. As data owners, nearly all RIs are employing their own data protection measures and possess a legal framework that forms the basis of access and data usage rules [16, 17]. But with the BioMedBridges project that connects ESFRI RIs data sharing will go beyond the capacities of individual infrastructures to ensure efficient and comprehensive integration of data and data services across different RIs and different research domains. Thus, the development of data bridges has led to additional needs for legally interoperable data sharing [18]. Moreover, RIs are supposed to create a new research environment in which researchers can make use of shared access to data, data services, and computing facilities regardless of the type of data involved and their location. For such a new research environment issues of data protection and data governance must be addressed.

But the heterogeneity of policies for data access between different data providers and the lack of national harmonized legislations in Europe have increased the relevance of legal interoperability as a key aspect of research collaboration [18, 19]. Researchers are confronted with the question, whether, on what basis and with what limitations, human data can be used freely and made available to support open research and open science. Human data combined from multiple sources with different usage restrictions and rules may result in combined datasets that inherit the restrictions and usage rules imposed by each source. In such cases, the most restrictive usage rights from any of the sources may apply. Because of the higher identification risk of the combined data sets stronger restrictions for using combined datasets than the most restrictive rights from any of the sources alone may in some cases be necessary.

The research community in general and BioMedBridges in particular want to support approaches and methods for open data and open science [19, 20]. In view of the mentioned challenges and restrictions associated with the processing of human health data, we searched for ways to present researchers who access databases, share or link data, with the requirements for legally compliant data sharing with as little restriction on research and as little legal pre-knowledge as possible. We developed an integrated approach that considers data protection as well as data ownership (IP and licences) and that focuses primarily on the requirements for legally compliant data sharing. In order to employ a pragmatic approach that bypasses the intricacy of legal discussions we turned to methods of IT requirements engineering and applied concepts of requirements engineering to legal problems to create legal requirements clusters. Requirements engineering is the systematic process of developing requirements and traditionally, it represents the first phase of the software life cycle in which the functional and non-functional requirements to be met by the system are elicited and documented [21]. Because scenarios have been advocated as efficient means of improving requirements engineering [22, 23], we used scenarios of different data bridges to elicit requirements. Usage scenario descriptions of data bridges formed our basis for the development of requirement clusters for data protection, data security, intellectual property (IP), biosamples and animal protection. These requirement clusters define conditions under which the diverse data bridges can be used conformant with regulations and rules. These rules and regulations for access, processing and transfer of human data were collected, listed, and interpreted. Finally, we developed an interactive tool to query the developed knowledge base.

## Methods

### Analysis of the legal landscape for data sharing

**Fig. 1:**
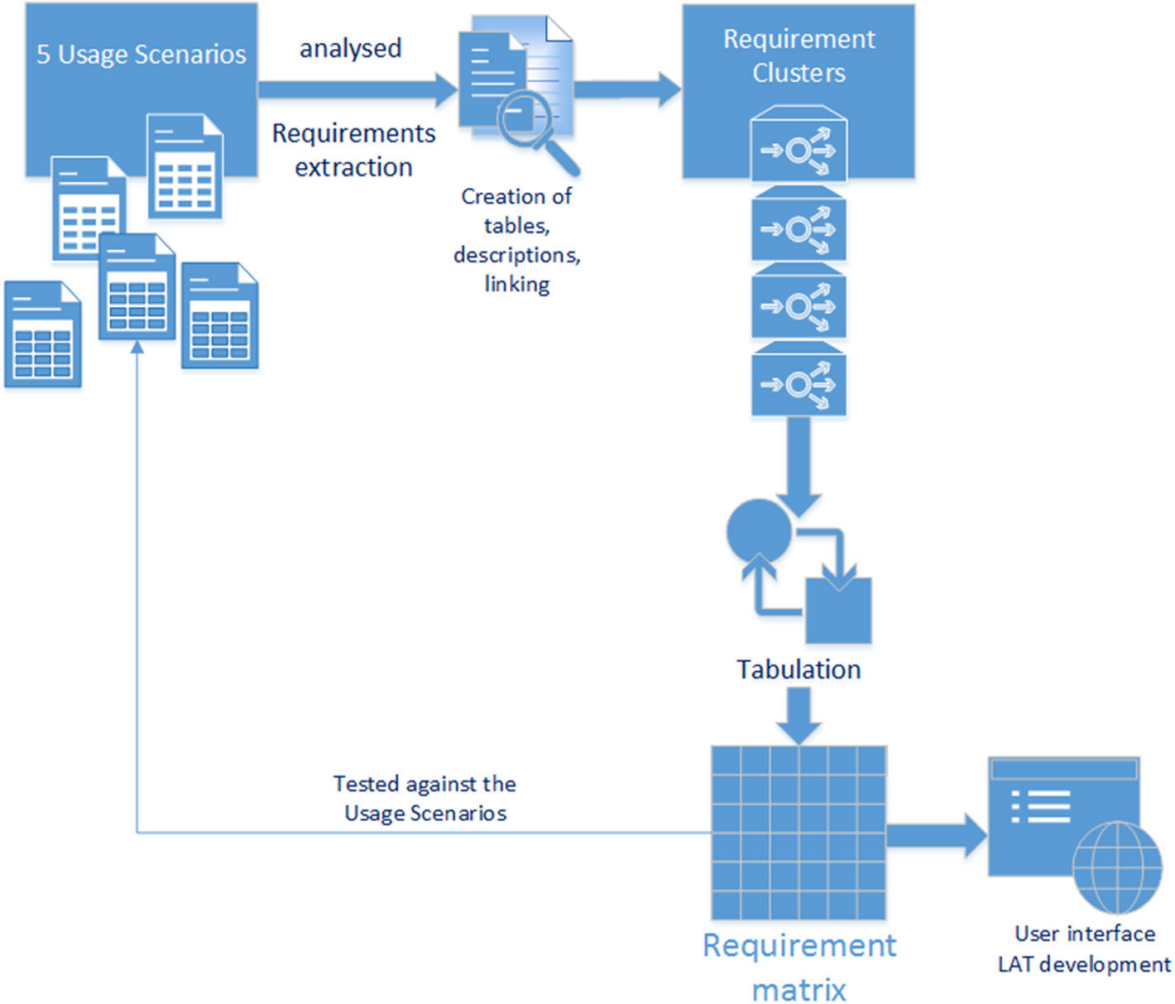
Creation of the Requirements Matrix. Extracted rules and policies, data types used and combinations of access rules and the results of the analysis went into the creation of the matrix. Using tabulation the matrix was created, whereby the requirements are listed as rows and the columns represent data characteristics and applicable rules (e.g. data subject, data type, data protection (identifying, pseudonymous, anonymous), purpose of data processing, legal approval / consent, etc. The values of the different cells contain the extracted rules for certain data types and for certain combinations of data types and their possible combinations

We implemented a novel approach by using concepts derived from computer science, especially from requirements engineering, which were adapted to define and collect legal requirements for the design of data bridges. In this context, we defined “legal interoperability” as an extension of the general interoperability concept for diverse systems that forms conditions where diverse rules sets allow the exchange of data between them. We conceptualised the planned data bridges as “interfaces” between data sources for such data sharing. A “legal interface” [24] is characterized by “requirement clusters” defining applicable rules, roles and policies used by database owners (data controllers) and acting as a kind of filter between different data sources to allow for compliant data transfer (Fig. 1).

As a first step to understand the underlying basis of all data access and processing rules, the legal landscape for data exchange for research purposes was analysed, and it became very soon clear, that it would be impossible to cover all legal details from every European country involved in the data bridges. Thus we decided to focus on the European data protection framework consisting of EU Data Protection Directive (Directive 95/46/EC) [25], and Good Clinical Practice (GCP) [26] with regard to data protection and the WIPO (World Intellectual Property Organization) framework [27] with regard to intellectual property / license rights.

In the next step, access and usage rules of important biomedical databases (e.g. Amigo, ChEMBL (chemical database of bioactive molecules with drug-like properties), Ensemble, Gene Bank, European Genome-phenome Archive) were evaluated to determine their conditions of use, any restrictions, the existence of licenses, the role of the data controller and possible involvements of data protection committees or similar boards. These rules were compared with applicable legal regulations. Because we interpreted data bridges as interfaces that allow transitions of data flows between data providers with different "policies" and “data use agreements”, combinations of all employed data sharing conditions and requirements between involved databases were analysed including the requirements for data sharing by all data providers of our usage scenarios (Fig. 1). Though, this covers nearly all databases with a high number of users, many minor and peripheral databases, though important for a specific research field, were not considered.

At first we attempted to use an ontology to represent the applicable access rules and privacy requirements for data sharing. Semantic technologies are used to address many challenges relating to privacy and data protection, even providing the basis for reasoning over domain knowledge for decision support in the area of privacy compliance [28]. Nonetheless, in our experience, ontologies are still not suitable to represent the complexity and intricacy associated with many of the rules and combination of rules we encountered. We had to take another road by employing concepts from requirements engineering.

### Usage scenarios were used as basis for the creation of legal requirement clusters

Our approach of integrating domain experts of the use cases to develop the usage scenarios guaranteed effective requirements elicitation on the basis of the actual characteristics of the data sources and the kind of data processing involved in different data bridges. Usage scenarios that represent different data bridges were created by developing data flow descriptions for the Image data bridge, Phenotype bridge, Personalised medicine data bridge, Structural data bridge, and Biosamples data bridge (Table 1). We created data sharing scenarios because scenarios as a technique are increasingly used as a requirements discovery tool. A requirements scenario is a simulation of what happens within the boundaries of a specific scene for the purpose of discovering business requirements [23]. A more formal and restricted representation of a scenario is the use case. A use case describes a scenario as interactions between an actor and a system and describes requirements, preconditions, triggers, activities, etc. We used the term "usage scenario" because our scenarios describe processes as well as requirements for the sharing of data between RIs and we used terminologies suitable for this activity (e.g. actor, databases, legal requirements, access type). By using use cases instead of scenarios we would have been restricted to descriptions where always the user is outside the system and the system central, a description useful for software development, but less for legal requirement clusters. For all five usage scenarios data sources (databases), involved actors (e.g. investigators performing analysis, bioinformaticians, data source owners, owner of mouse line) and requirements/prerequisites for the corresponding data bridge (requirements for the provision and sharing of data) were collected. In addition, descriptions of data exchange processes, events and actions (data access and processing procedures associated with the data bridges), and involved data types and data standards were addressed. For each data provider, the available data types, descriptions of the mode of access (open, open restricted, restricted), data standards, as well as the linking of databases (what data are linked or aggregated and how is the linking done, e.g. through phenotypic annotations) and the transfer of data (for a specific data provider, what types of data and metadata are transferred) were gathered. The scenarios are completed by descriptions of the added value of the created data bridge. In principle, our usage scenarios served as context for the specification of the legal and ethical requirements [29]. In several iterative rounds of discussions and revisions, data flows and data processing procedures in all five usage scenarios were analysed for applicable regulations and rules. We were supported by members of the different BioMedBridges use case groups. Finally, the results were reviewed by internal reviewers and by legal experts.

**Table 1:** Five usage scenarios

Data bridge	Description
Image data bridge	A data bridge that facilitates the comparison of cellular phenotypes specific to individual genes with morphological imaging data from diseased tissue specimens, from both human and mouse tissues.
Phenotype data bridge	Datasets from mouse or human are collected that relate to the disease states of diabetes and / or obesity. After the annotation of these datasets, a service allows the automatic identification of phenotype matches across mouse and human.
Personalised medicine bridge	The data bridge enables access to and integration of, often heterogeneous and dispersed, patient data to enable better treatment decisions for individual patients by using a data analysis informatics pipeline.
Structural data bridge	A data bridge connecting databases with structural data and protein interaction data. Researchers receive access to a range of available structural techniques, such as crystallography, Nuclear Magnetic Resonance (NMR), MS or EM, and will be presented with a comprehensive structural model.
Biosamples data bridge	Researchers receive information about available sample data through the BioSamples database with the aim to set up clinical/translational research collaborations.

The five usage scenarios representing different kinds of data bridges used to create requirement clusters and to evaluate the developed legal interfaces

All requirements were screened against descriptions of the usage scenarios to verify the results (Fig. 1). In all cases during the screening process, the most important criteria for the application of the requirements turned out to be the differentiation between human and non-human data. In addition, the location of the data provider and the type of data to be provided played key roles. In our scenarios the data provider is one of the databases in a research infrastructure that offers services like the download of data to a researcher. In case personal data is involved in this data sharing national data protection regulations may apply and the location of the data provider has to be considered. Though, the location of the data object, the human source of the research data, is also of importance for compliant sharing of personal and sensitive data, consideration of the rights of the data object belongs to the role of the data provider and is integrated in his rules and policies.

Based on the extracted requirements, eight tables were created containing structured descriptions of data protection requirements: Requirements for animal data and human data, potential identification risk for human research subjects, pseudonymous human data, anonymous human data, requirements for getting access to data/biosamples, requirements for linking and sharing restricted access data and open access data, IP requirements, and requirements concerning the security of biosamples (Table 2). The requirements for animal data were not further pursued, since they raise no data protection issues. The created requirement clusters define conditions under which different data access and data sharing activities can be used conformant with regulations and rules. All requirements are based on the assumption that all data providers employ rules and policies that are compliant with the relevant national and European laws and regulations.

**Table 2:** Requirement clusters

Name of cluster	Requirement tables	Description
Data protection/privacy		General data protection requirements applicable to data bridges; conditions, which must be fulfilled in order to legally process personal data.
	Table 1 Requirements for animal data and other data	Requirement in relation to: • Deletion of personal data. • Anonymising of personal data. • Obtaining consent from the research subject / researcher
	Table 2 Potential identification risk for human research subjects	Requirement in relation to: • Removing metadata • Removing data on an image • Altering the image • Link between biosamples and data has to be protected. • Identification risk has to be checked after data merging • Re-identification risk based on genetic data.
	Table 3 Pseudonymous human data	Requirement in relation to: • Obtaining informed consent from the research subject • Checking current Informed consent. • Right to be informed
	Table 4 Anonymous human data	Requirement in relation to: • Verifying the re-identification risk
Data security		Data security issues with a focus on access control; measures to protect data from possible outsider attacks, as well as from re-identification attempts
	Table 5 Requirements for getting access to data/biosamples	Requirement in relation to: • Data Access Committee Approval • Research Ethics Committee Approval • Renew Consent • Anonymising data • Material Transfer Agreement
	Table 6 Requirements for linking and sharing restricted access data and open access data	Requirement in relation to: • Different Access Tiers • Authentication/Authorization system • Audit trail • Secure data transfer (e.g. via encryption) • Approval for the use/processing of data • Approval for redistribution/sharing • Identifying data/personal data should be stored separately • Limitation of use • Removal of data • Regular backup of database
Intellectual property and licences		Prevention of the infringement of intellectual property rights needs to be fulfilled in order to protect intellectual property rights within data bridges
	Table 7 Overview of the IP requirements cluster	Requirement in relation to: • Data and metadata encryption • Data access agreement • License agreement • Limited liability statement • Material transfer agreement • Non-disclosure agreement • Disposal of biological samples/material • Removal of identifying metadata • Temporary embargo of data sharing • User authentication • Data labelled as 'restricted' • Staff awareness
Security of biosamples		Security issues concerning biobanking, measures that have to be taken to securely use and share biosamples
	Table 8 Requirements concerning the security of biosamples	Requirement in relation to: • Delete personal/identifying data • Renew consent • Get specific consent • Get broad consent • Get Research Ethics Committee approval • Approval from a relevant regulatory body/authority (concerning biobank research) • Data Access request • Negotiate Material Transfer Agreement • Anonymisation of data • Approval from a relevant regulatory body/authority responsible for data transfer • Data Access request • Personal and Identifying data should be stored separately • Remove researchers’ personal data • Ask researcher to publish personal data

Five requirement clusters were created containing eight requirement tables connecting requirements with constraints and protection measures

In the next step, the extracted requirements and rules of the requirement clusters form the core of a single requirement matrix.

### Evaluation of the requirement clusters and creation of the matrix

**Fig. 2:**
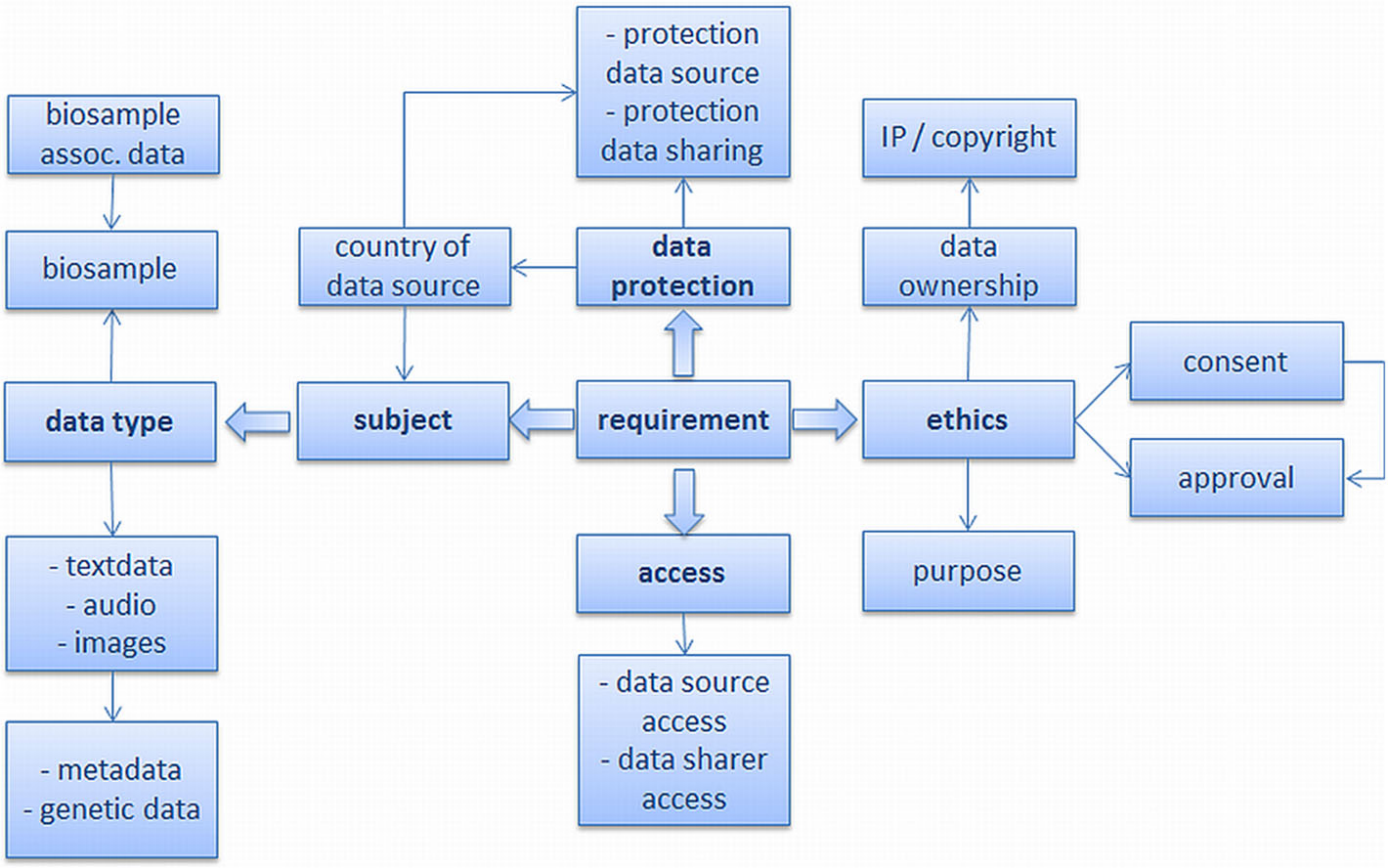
Dimension tree of the requirements. The criteria of the dimension tree organise requirements derived from five requirement clusters

**Fig. 3:**
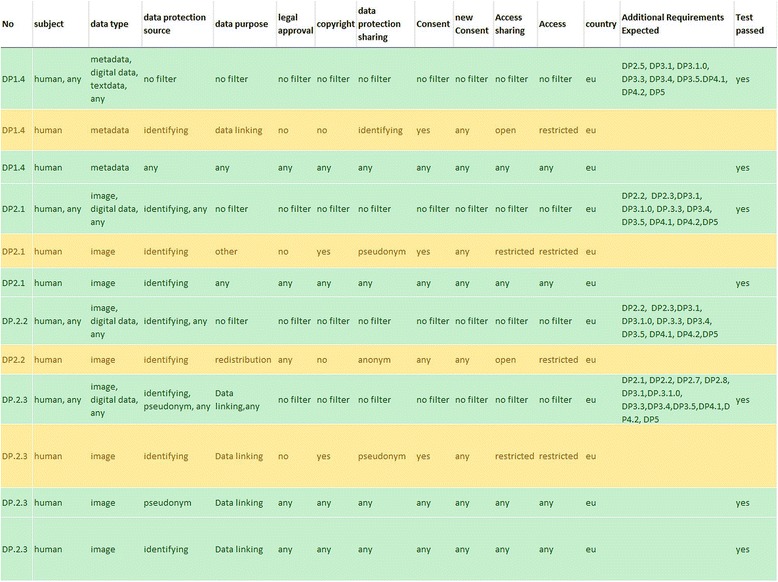
Result of the semantic testing of the requirements matrix. Part of the MS Excel sheet representing the requirement matrix is shown used for filtering the values of the requirement dimensions. Requirements were validated by calculation from the matrix using filtering

Our aim was to combine the information of the requirement clusters in a way that it becomes usable for researchers faced with a compliance problem. The tables of the requirement clusters are already arranged in a way that requirements are collated to specifications and conditions for compliant data sharing (e.g. data subject, data type, anonymous, pseudonymous). But we needed more specific terms to organise the multitude of requirements. Based on an analysis of usage scenarios and requirement clusters we determined conditions when requirements can be successfully applied and formulated these as dimensions of the corresponding requirements. For this purpose, different conditions of a requirement were defined that determine when a requirement can be employed (dimension). To define these dimensions the most important conditions from the requirement clusters, data type and data subject, were used and specified by text data, audio data, metadata, genetic data, biosample data, and country of data source. The additional dimensions Data protection (protection of data source, protection of data sharing); Access (data source access, data sharer access) and Ethics (purpose, consent, approval, data ownership and IP/copyright) completed the specifications and formed an interrelated dimension tree (Fig. 2). Requirements and dimensions were combined in form of a matrix; for each requirement listed in a row the corresponding dimensions (columns) obtained a value indicating if and how the requirement applies. All possible combinations were tested and compared with the results of the requirement clusters. In this way specifications/conditions and requirements were hard-wired. Because of their importance specifying data protection needs, the data subject, ethics (consent and approvals), the country of database location and the data type were placed in the centre of the matrix and became in a later step the first questions in the LAT survey. The dimension “data protection source” of the matrix indicates if data is identifiable or not; it classifies the stored data of the data source as: anonymous, pseudonymous, or de-identified data (Fig. 3). Two types of data access were considered; access to the data source is open access, or access is restricted. In addition, the type of access desired by the user for his/her data with values for open access; restricted access, authentication/registration, and combined access were allowed.

The requirements matrix is the basis for the assessment activity of the LAT. By answering the questions of LAT, relevant dimensions are selected, which are represented as columns in the requirements matrix and are mapped to associated requirements. In this way, explanations and guidance provided by LAT are generated on the basis of the requirement matrix; they are dynamically loaded from the database. LAT can generate the relevant requirements that belong to a specific selection of user specifications. In detail, the columns serve the data protection criteria (purpose restriction, need for approval, need for consent, form of access (open, restricted) and kind of copyright required). The rows are the requirements depicted as combinations of values that are connected by “and” and thereby linking specifications, like “pseudonymisation”, “anonymization”, “identifying”, “any”, “no” together to come up with a rule that is expressed in the LAT as a recommendation for data sharing. Because the matrix generates results for all kind of data including animal, structural and human data, it creates for animal and structural data the value for the data protection of “no”. The matrix also considers the case that non-human data may be associated with identifying metadata (requirement No. 1.1–1.3).

For example, whereas requirement No. 2.1 contains human imaging data with identifying information at the data source used for any purpose (including data linking), requirement No. 2.7 is the same but contains human imaging data where identifying information has been exchanged by a pseudonym. Therefore, the corresponding data protection column indicates “any” as value, representing the data protection measures (necessity for pseudonymisation, anonymization, and additional constraints) in the first case and “anonymous” in the second case. The rational for this mapping is that pseudonymous data when linked to open or other pseudonymous data are subject to an increase in the risk of data subject identifiability. This argumentation shows that to some degree value judgements and interpretations are inherent in the matrix. The reason for this is that in general legal terms and statements require interpretation. Data protection acts often do not define terms, like “anonymization”, “consent”, “re-identification” and “research purpose”. For example, genetic data can cause the problem that despite comprehensive anonymization the re-identification of a person may still be possible if relevant additional knowledge exist in other databases. On the other hand, for certain images the potential risk for face recognition in 3D reconstructions magnetic resonance imaging (MRI) exist. For building the matrix, the definitions of terms like anonymisation, pseudonymisation, identifiability, etc. were based on the ones data providers are using and are transcribing into their access and usage rules. Nonetheless, when combining different rules and policies due to data sharing or linking of data to other data sources, we made the inherent judgement that when two different rules have to be combined, the stricter rule should apply and it should be considered that linking of data may increase the identifiability of data subjects. In order to support legally sound data sharing, data processing agreements and similar documents are often needed. Contractual templates with generic texts were developed and integrated into the LAT. These templates and text building blocks address some major issues concerning trans-border data sharing for research. The legal background, however, is far from being clear and reliable; it is undergoing a process of being established, changed and harmonized throughout Europe. Therefore, the templates are based on a survey of a number of European and national contractual models and forms; however, these are in no way the only possible solution but suggest certain policies and assume certain legal opinions that seem to be currently prevailing. This situation was made clear by providing several explaining texts, such as “how to use the tool” and “how to use the template”. In addition, some of these texts point to major legal debates such as “Can biosamples be anonymised?”

### Technical realization of LAT

**Fig. 4:**
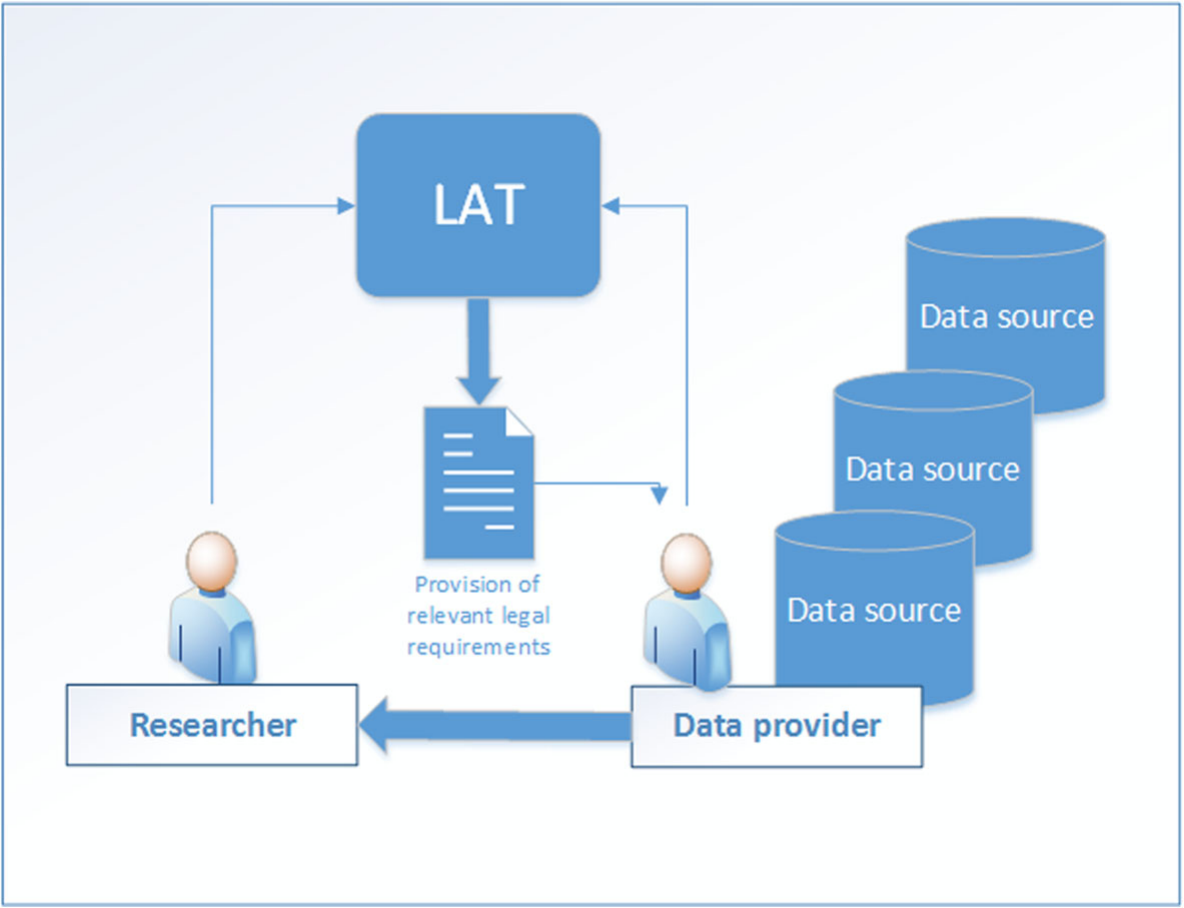
Usage of the LAT. The user answers the questions of the LAT and receives a list with relevant requirements for data access and data sharing

To access and query the knowledge base a user interface was created. This user interface enables the querying of the requirement matrix by researchers without any previous legal knowledge. The main aim was to create the user interface as easy to use and as intuitive as possible. By answering questions about the kind of data involved, the user specifies conditions for applying the requirements (dimensions of the requirement matrix) and obtaining the applicable requirements for a specific data provision or sharing situation (Fig. 4). LAT provides researchers with a set of requirements to pay attention to, as well as regulations to consider, and aspects to watch (e.g. increased risk of identifiability). In summary, LAT gives no legal advice, but provides requirements for different kinds of data sharing situations to enable legally compliant data exchange. The technical realization of LAT employs various open source components (Table 3). LAT has been developed as a plain html web application containing the JavaScript frameworks AngularJS and jQuery. Spring MVC and Hibernate frameworks were used to realise the backend implementation.

**Table 3:** Technical components of the LAT tool

Tool	Version	Licence
Ubuntu	12.04.5	GNU GPL
Apache Tomcat [65]	7.0.42	Apache License 2.0
jQuery	2.1.4	MIT License
Hibernate	4.3.11	LGPL
Spring MVC	4.1.6	Apache License 2.0
AngularJS	1.4.3	MIT License
Java [66]	1.7.0.72	GPL Version 2

Components of LAT tool and contribution of Open Source Software and their licences

### Testing and evaluation of LAT

The complexity of the requirements matrix made it necessary to test all possible queries and assessment results in a structured way. The assessment tool was tested on two different levels: first, semantic and logic integrity of the requirements matrix; second, correctness of the implementation (tool). To test the requirements matrix, two test scenarios were developed: first, a non-contradictory requirement test (it was separately tested whether requirements that were assessed resulted in contradictions and whether all compatible requirements were selected); second, it was tested whether queries resulted in the correct requirements. Two checkers worked separately and exchanged their tests in order to cross-validate their results. All tests were performed with the help of the filter tool of MS Excel.

**Fig. 5:**
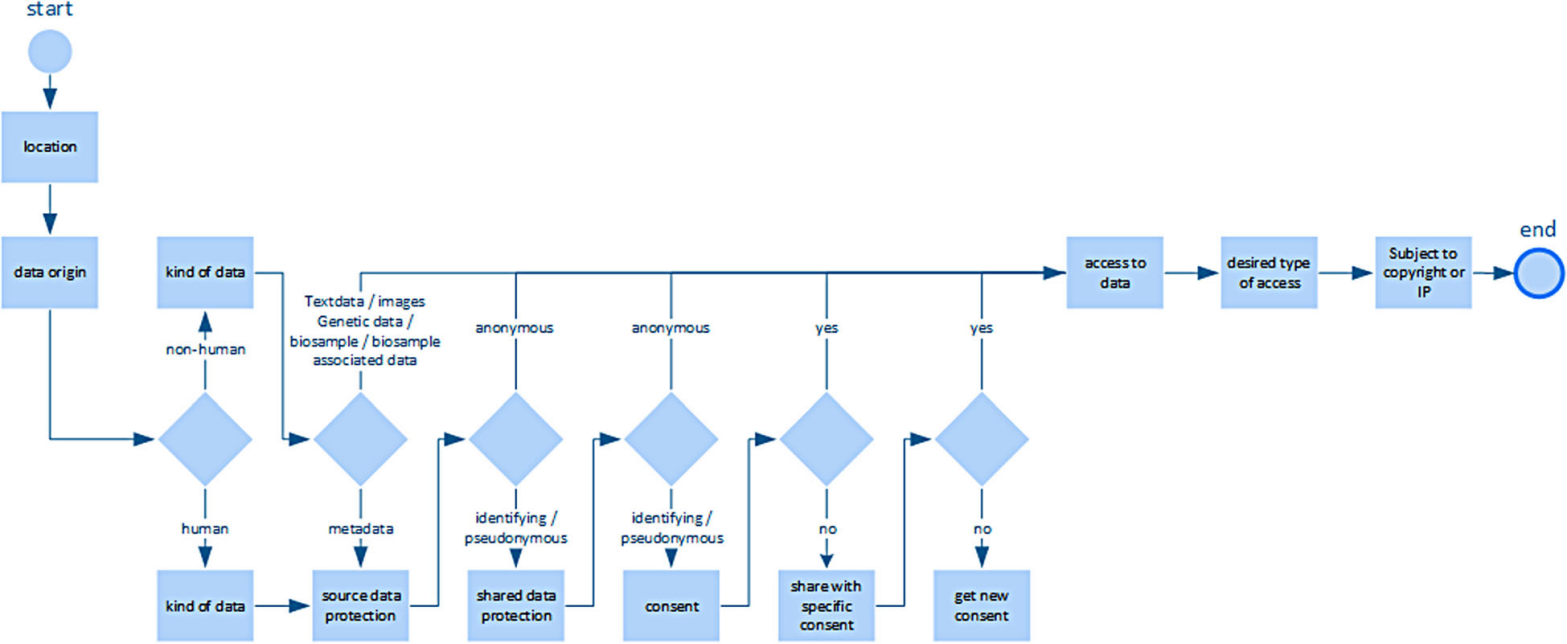
Course of a generic query. Shown is the workflow through the questions and decision points that characterises the user interface of LAT. The workflow begins top left with “location of data source” and ends at the right end. Depending on the data source not all of the specifications need to be determined/answered. Simultaneously with answering the questions, relevant results are presented (bottom lane)

After completion of the matrix test, the matrix was frozen and the tool testing was begun. It was tested if the query workflow precludes all non-feasible selections (Fig. 5). For this purpose, a query use case was developed and expected requirements were calculated from the matrix by filtering (Fig. 5). Then the validated queries were entered into the tool and the results were compared with the expected ones. These tests showed that the requirements matrix was implemented correctly.

In addition, a preliminary usability testing was performed. Three interviews with potential users that were members of the BioMedBridges consortium were conducted. The potential users were typical researchers, working about personalized medicine at FIMM (Finland Institute for Molecular Medicine) (Finland), about population cohort studies at the THL (National Institute for Health and Welfare) Biobank (Finland) and as a biologist at the Institute of Experimental Genetics (Germany). The tool could be used by all evaluators without problems. No issues with the user interface were encountered. LAT was seen as very useful for the topics of data protection and / or ethics during data sharing. As strong point it was mentioned that the tool is able to provide assistance and guidance in the case of uploading of identifying data to an open access data source. It was stated that the tool should be able to assist in the planning of clinical studies, e.g. by simplifying the right choice of informed consent for different settings. Though users had different opinions about the guidance function of the tool, checklist for different types of IP issues and informed consents and recommendations for means for efficient data anonymisation procedures were asked for. Meanwhile LAT has been demonstrated and evaluated during several workshops of the BioMedBridges project.

### Provision of the tool

LAT is made available under an Open Source licence (Apache) upon request. Access to a fully functional test version is provided [30] as well as a training website with links to documentation [31].

## Results

Our methodological approach created rich information about requirements for compliant data access / data sharing by studying conditions, specifications and relations for data sharing based on actual research data flows and databases used for research and by considering rules and regulations. Representing the core of the knowledge base the requirement clusters are presented in the following text in detail. Table 4 gives an overview over the involved databases and the main access rules of the different requirement clusters; detailed listings of the requirements can be found in the Additional file 1: Tables S1–S5.

**Table 4:** Databases and their access policies considered for the requirement clusters of data bridges

Data providers	Summary of main access restrictions and policies
Requirement cluster: Imaging bridge	See: Additional file 1: Table S1
Mouse tissue imaging data (Infrafrontier), Human tumour tissue data (BBMRI/ FIMM), MitoCheck (cell-based RNAi screens), WebMicroscope (mouse and human image data sets), Ensembl, ArrayExpress, Phenotator, MitoCheck	Restricted access, data linking only possible, if the data provider gives permission based on the availability of informed consents, consent form which permits such a research may be required, application to the steering committee or principal investigator, Images are owned by image generator
Requirement cluster: Phenotypic bridge	See: Additional file 1: Table S2
EuroPhenome, IMPC, Gene Expression Atlas (GXA), ArrayExpress, ChEMBL, Metabolights, Reactome, CERM datasets, Biobank/BBMRI (University of Graz, Austria)	Predominantly open access, open restricted for private data (pre-publication/ unpublished), open access, when data is used for research purposes, biobank with restricted access (access rules include project application and approval committee)
Requirement cluster: Personalised Medicine bridge	See: Additional file 1: Table S3
ICGC (International Cancer Genome Consortium), TCGA (The Cancer Genome Atlas), EGA (European Genome-phenome Archive), Cosmic (Catalogue of somatic mutations in cancer), GEO (Gene Expression Omnibus), Array-Express, ChEMBL, Reactome, Ensembl, Drugbank, Pharmgkb, BioSD, Biobanks (BBMRI), EU-OPENSCREEN, ECRIN (CTIM), FIMM Institute for Molecular Medicine Finland (EATRIS)	Open access and data with and without restricted access, different policies apply, controlled access datasets means access control, Data Access Compliance Office (DACO) handles requests from scientists for access to controlled data, requirement for user certification via Data Access Request, download of datasets must be approved by the specified Data Access Committee (DAC), requires users to sign a Data Access Agreement (DAA), which details the terms and conditions of use for each dataset, all controlled access downloadable datasets are encrypted, restricted access usually for pre-published/unpublished data
Requirement cluster: Structural Data bridge	See: Additional file 1: Table S4
UniProt, AmiGO (Gene Ontology database), EMDB (Electron Microscopy Data Bank), IntAct (Molecular Interaction Database), GenBank (NCBI), ELIXIR, BMB database	All are open access
Requirement cluster: Biosample data bridge	See: Additional file 1: Table S5
	Mainly restricted access. Open access for aggregated anonymised information or metadata about samples, for data access researcher has to apply to Data Access Committee (DAC) of biobank and has to agree to use data only for research, which has been specified between biobank and researcher, and not to try to identify patients

### Requirement cluster: imaging bridge

The analysis of cell and tissue image datasets and the linking of their associated annotations and metadata can provide powerful predictors for biomarkers as well as for drug targets in cancer. In this usage scenario four major databases were considered (Table 4). Though heterogeneous in data content, their data protection requirements are rather similar: MitoCheck database, Ensembl, and ArrayExpress for cancer expression data, contain anonymous human data that is stored in open access databases. On the other hand, the Mouse Cell Line database restricts its access.

### Requirement cluster: phenotypic bridge

The Phenotypic data bridge addresses the challenge of connecting different ontological phenotypic annotations of mouse and human in the domains of diabetes and obesity. This data bridge is characterised by the involvement of a large number of open access databases (Table 4): EuroPhenome / IMPC (International Mouse Phenotyping Consortium), ArrayExpress / Gene Expression Atlas, ChEMBL, Metabolights and Reactome. CERM (Centro di Ricerca di Risonanze Magnetiche) data sets are defined as metabolomics data (only selected datasets will be used) and are stored in a database with restricted access. The datasets of the University of Graz contain mouse and human data and are stored under restricted access conditions.

### Requirement cluster: personalised medicine bridge

Personalised medicine uses personal data sets that can identify the corresponding data subject. Therefore, research in personalised medicine needs special protection. Anonymous data is available in published articles, and certain databases: Cosmic, ICGC (International Cancer Genome Consortium) (in parts open), TCGA (Cancer Genome Atlas) (in parts open), ArrayExpress, GEO (Gene Expression Omnibus), Drugbank, ChEMBL, Pharmgkb. ICGC, TCGA, EGA (European Genome-phenome Archive) are databases with restricted access (Table 4). In addition, personal data stays in the protected environment of FIMM and is enriched by data from many different open sources.

### Requirement cluster: structural data bridge

A structural data bridge connects structural biology datasets from e.g. mass spectrometry (MS) and electron microscopy (EM) with specific features of biological systems, such as protein interactions. It provides links to open databases, such as GenBank, UniProt, AmiGO, EMDB (The Electron Microscopy Data Bank) (PDBe, Protein Data Bank in Europe,) (Table 4). Primary experimental data are stored at the INSTRUCT Centre, mostly in a local database. The linking of data sets is based on the availability of proper identifiers, e.g. proteins IDs.

### Requirement cluster: biosample data bridge

The BioSamples (BioSD) database at EBI is becoming an important resource for researchers to query the availability of biological samples from a wide variety of different biobanks. Biobanks follow their national and local regulations with respect to data protection and biosample management. Only information that can be openly accessed in biobanks is exported to and collected by BioSamples (Table 4). The access to BioSamples is open; no sensitive information on human subjects is provided or stored.

To ensure the simplicity of the tool, it was decided to concentrate on the requirements of the data provider role. This was in line with the “Ethical and Governance Framework” of BioMedBridges [32] that states that the data controller who is sharing/providing data is responsible for clearing legal requirements on the way to making the data available. Any constraints arising for example from consent limitations have to be reflected in data transfer agreements. In general, data can only be made available to the extent that is allowed under the local legal regime including ethics votes and patients’/donors’ consent. Because of this simplification, LAT can serve as a tool for data controllers, who want to get an overview over the legal constraints before making research data available to other researchers or within a research project.

### Development of a user-friendly interface for LAT

**Fig. 6:**
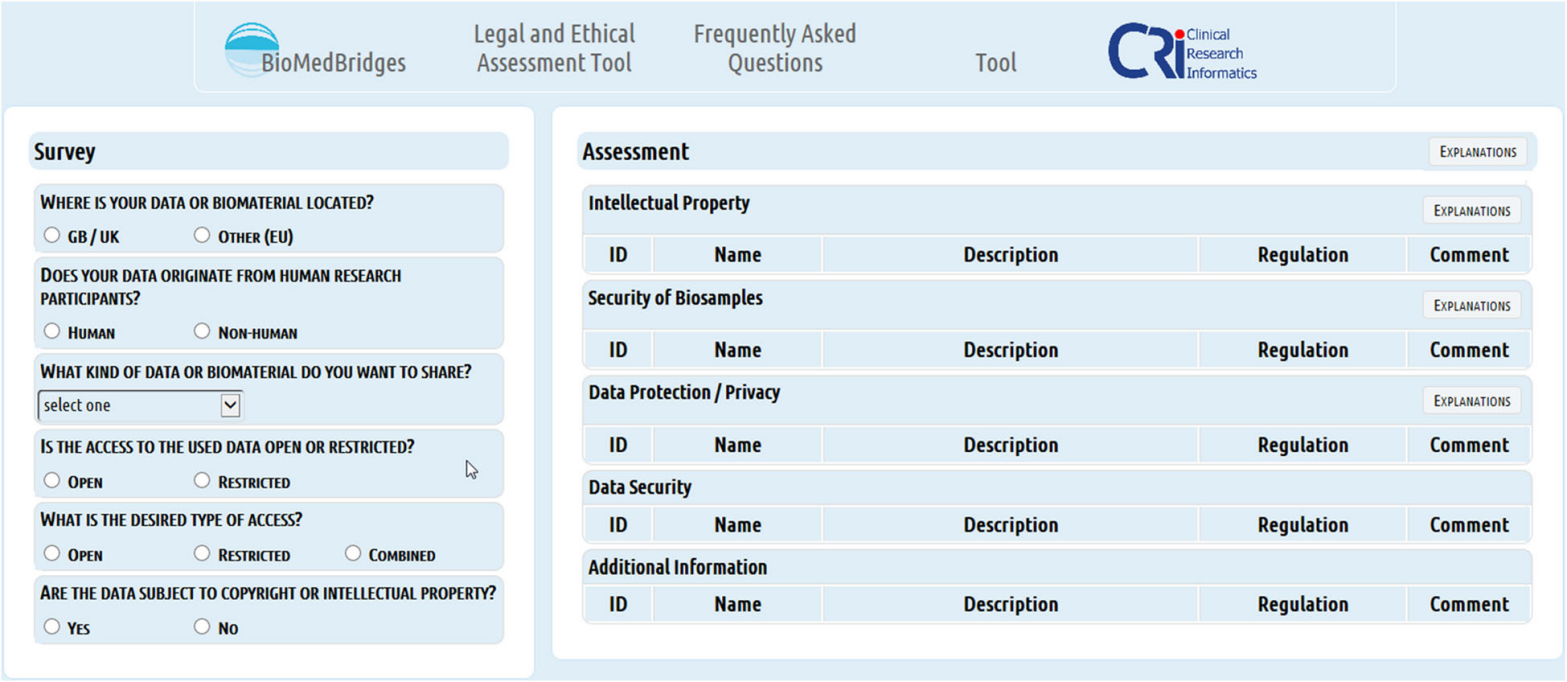
LAT interface consists of two parts: a survey part showing a set of questions (left side) and the corresponding results part with the assessment (right side). Because no question has yet been answered, the assessment part doesn’t show any results

We wanted to create a tool that is easy to use and fast in providing results. It should be used by the normal researcher and data provider, without having to resort on legal expert knowledge and the interpretation of legal texts (Fig. 4). For this purpose, an interactive user interface was created to query the knowledge base that is based on a questionnaire (Fig. 6). Because the requirements matrix consists of a large table with requirements as rows and specifications/conditions, the so-called requirement dimensions, as columns with all fields harbouring values for the realisation of the requirements, the matrix forms an easily accessible database for querying. The LAT user interface depicts eleven questions about dimensions covering the type of data sources and the kind of data to be accessed or shared to guide researchers through the requirement clusters situated in the knowledge base. The location of the data source, the existence of human data, the kind of data and existing access restrictions of the data to be shared or provided are specified. By answering questions by clicking on suggested specifications, LAT initiates corresponding queries and the user interface depicts the assessment with recommendations to what to look at to ensure compliant data sharing (Figs. 5 and 6).

**Fig. 7:**
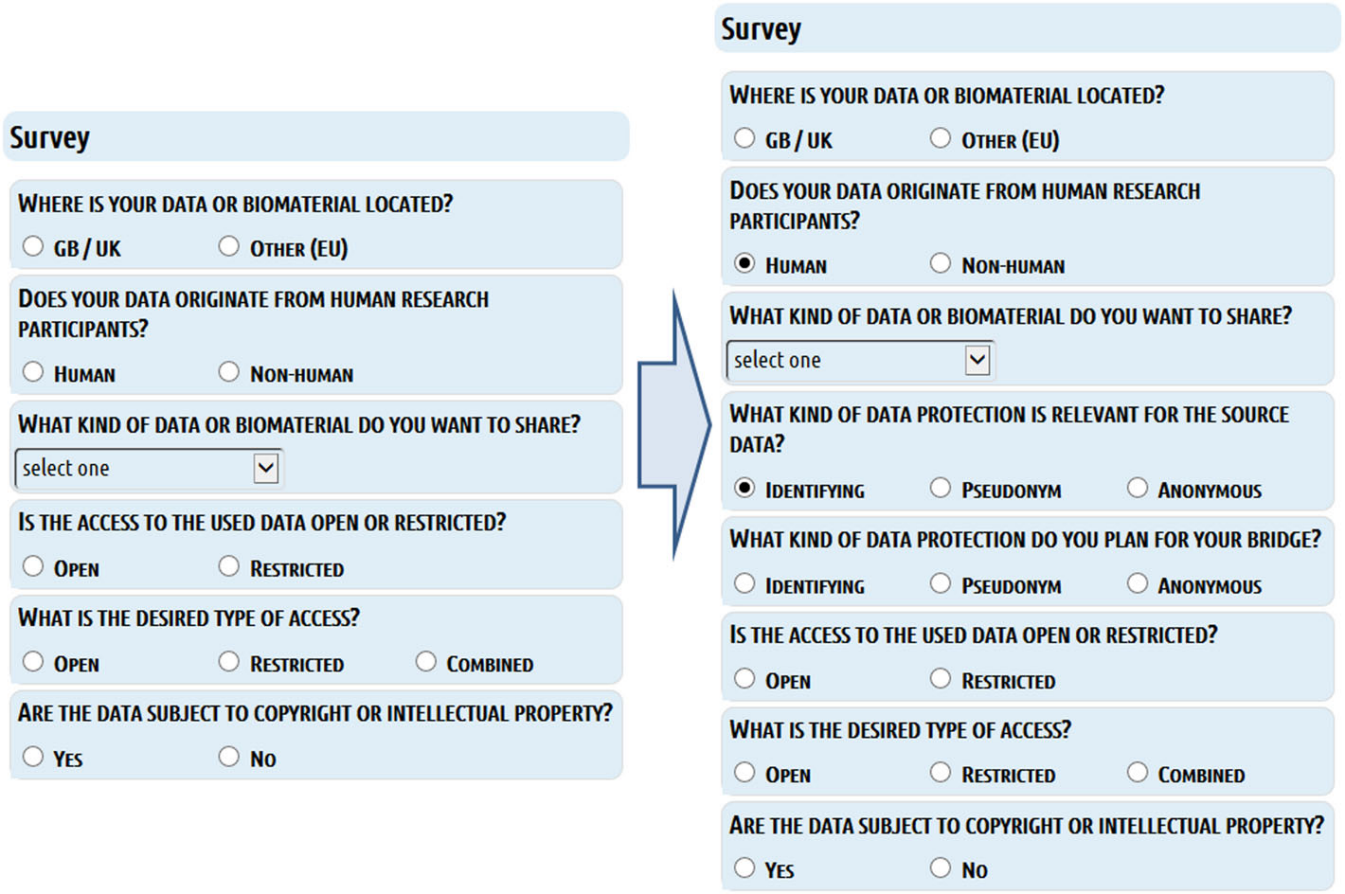
Conditional questions. Conditional questions depending on given answers. The example “HUMAN” data is shown. The right side shows the new, conditional questions

**Fig. 8:**
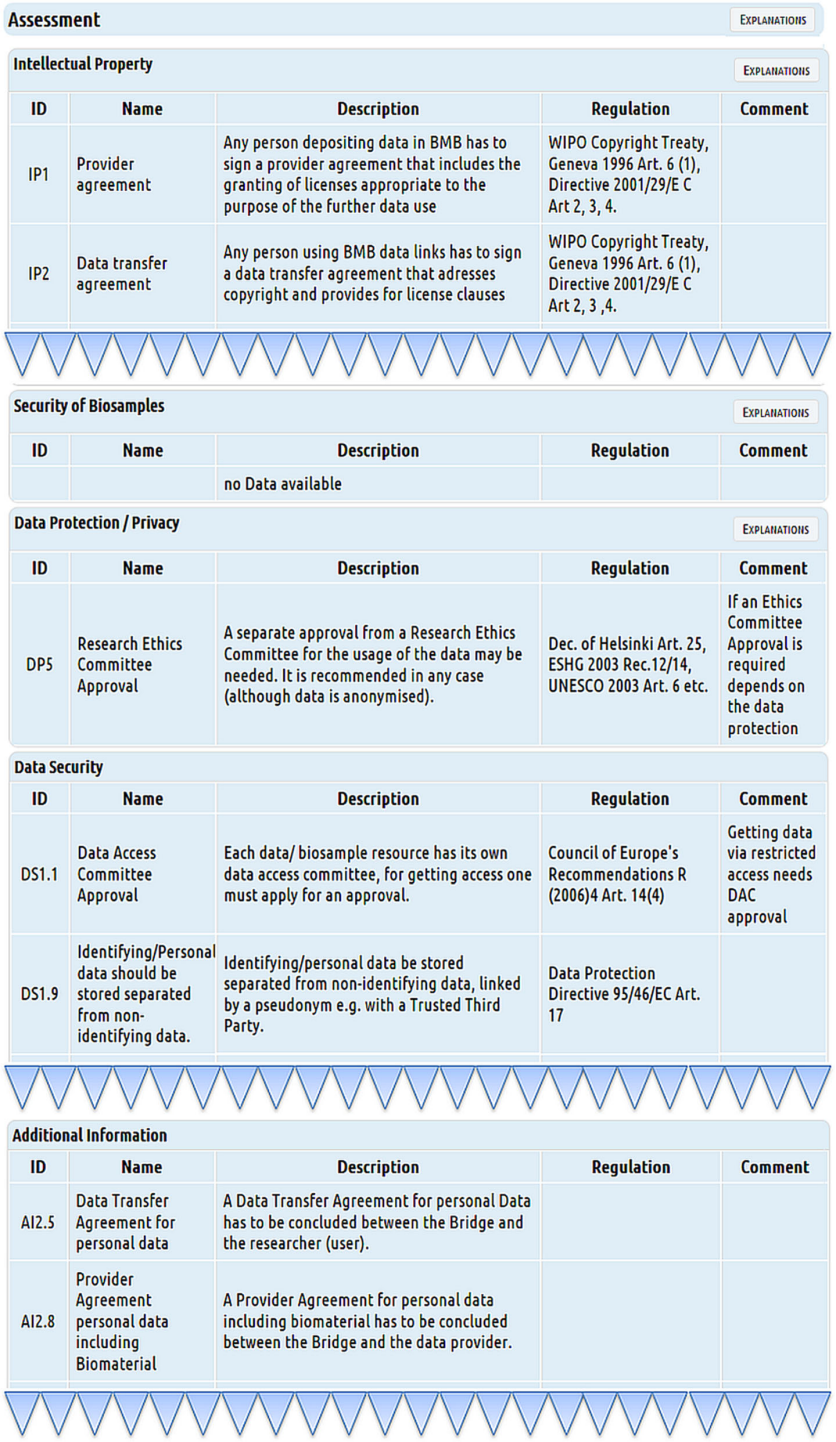
Exemplary assessment results. Only details of the lists of results for IP, Biosamples, Data protection, Data security and additional information are displayed. The upper parts of "Intellectual Property", "Data Security" and "Additional Information" are shown. The lower edges have triangulars to indicate that for the above mentioned topics more information is provided, but not shown in the figure. ID = requirement number. By clicking on the “Explanation” button, the user is linked to the respective regulations, or associated document templates, like Data Transfer Agreements

Technically, questions about the location of data sources, data origin (human, animal,…), kind of data, kind of access to data, desired type of access and the subject to copyright / intellectual properties are mandatory and lead to certain decision points (Fig. 5). Depending on the kind of databases accessed and the linking and processing of data planned, LAT responds to the answered questions, by offering different, new questions to be answered, thereby altering the workflow at the corresponding six decision points (Fig. 5 and 7). Simultaneously, with the choice of specifications, relevant results are presented on the same page. These results consist of legal and ethical requirements and recommendations that are specific for the selected kind of data and database (Fig. 8). The user interface provides supplementary online assistance in form of texts, like Q&A (Questions and Answers) and tool tips.

Depending on the given answers, conditional new questions may be presented (Fig. 7). Therefore, at the beginning of a query not all questions are shown and depending on the questions answered not every question possible may be shown to the user. For example, the selection of “HUMAN” as fundamental type of data triggers the display of an additional question about the kind of human data to be shared, a question allowing a more detailed selection between identifying data, pseudonymous data or anonymous data.

By answering questions and selecting specifications, the assessment part of the user interface is activated and presents results of the assessment as a list of requirements (Fig. 8). These results are specific for every given answer and give a direct output of necessary requirements as applicable rules and regulations for the specific data sharing case selected in the survey part. The results are presented as listing of requirements for compliant data sharing, covering data protection / privacy protection, data security, IP, biosample data security and additional information, like for example, the provision of sample texts for Data Transfer Agreements or Provider Agreements. Figure 8 shows an example of the sharing of human data with a restricted access database, where the assessment indicates that the requirements for the “right to know” of the data subject as well as the positive vote of a “Data Access Committee” may be needed. In addition, biosample data should be pseudonymised.

Templates for Data Transfer Agreements and Provider Agreements are available through the link “EXPLANATIONS”. As additional help for the user, next to the requirements in the results list, the corresponding regulations that form the basis for these requirements are shown. Links exists to supplemental texts explaining associated legal regulations.

## Discussion

When dealing with provision or sharing of human data, researchers are confronted with a lack of uniformity of legal requirements and differences in the use of legal terminologies. Different types of data usage licenses and additional restrictions on data sharing, like the processing of data only for non-commercial purposes, may apply. This complicated situation may discourage researchers to include human data to answer their research questions. Thus, the LAT was designed to provide researchers in an understandable and non-expert way with the basic requirements for their data access and sharing needs. We have collected and analysed data access rules, data usage rules and data provider policies of many of the leading European databases in the life sciences and extracted requirements from them. And we employed an approach originating from requirements engineering in the IT domain to the legal domain of data protection rules. Until now the legal domain is still confronted with problems to translate the legal logic embedded in texts of laws and regulations into machine readable rules; although some groups claim to be near to this aim [33–37]. Our first idea was therefore to use legal ontologies to transform legal rules into a machine-readable form. Several legal ontologies were analysed with respect to data protection, confidentiality, and intellectual property for usage in LAT. For example, the NEURONA ontology [38] is an application-oriented ontology and model for the knowledge necessary for the development of data protection compliance applications covering the correct use of security measures for personal data. OntoPrivacy [39] is a modification of a glossary of keywords from the Italian personal data protection code to support search and allow retrieval and visualization. The LRI-Core Ontology (Ontology of Fundamental Legal Concepts) [40] covers main concepts that are common to all legal domains. None of these ontologies was useful for us, though, because none covers data sharing for research purposes and none could cover the logical intricacies associated with different forms of informed consent and different definitions of anonymity. As mentioned above, the diversity and complexity of the rules governing data protection in Europe results in the need to abstract from these complex rules. Privacy requirements are the obligations that must be fulfilled for a compliant sharing and processing of sensitive patient data and this covers policies for consent, anonymity, and the right of the data subject to be informed. Such data protection policies play a major part in user specific privacy protection for service-oriented architectures [41]. In this architecture, privacy services (PS) are used in combination with privacy policies to create privacy contracts that outline what is allowed or not allowed with identifiable information. This approach will play an important role for service-oriented research applications. Another way is to employ ontology-based privacy compliances [41, 42]. The modeling of high-level policies derived from European and national data protection laws can create privacy-aware access control policies by using semantic Web technologies [43, 44]. But in the complex legal domain, the modeling of legal relationships cannot be expressed in OWL alone, because the logic of legal properties is not rich enough for this purpose resulting in the need for additional methods, like the use of the Semantic Web Rule Language (SWRL).

As a consequence, we decided to go the other way; our focus was not on the analysis and interpretation of legal texts, but on the application of the rules the different data providers require and provide. Our approach was to abandon the employment of ontologies to include semantic of legal regulations in our tool, but to concentrate on the access rules and data processing rules of European databases and extract the most important concepts in form of a dimension tree that links the data access requirements with the ones for data sharing.

But conciseness of approach always entails a limitation in the granularity of information and therefore, our tool has to leave certain questions open, which can only be resolved with the local authorities, like data protection authorities or ethics committees. Nonetheless, the user of LAT gets an overview over the major legal requirements and guidance, including the information when to consult legal departments or local authorities. Because the requirements matrix as a table is easily extensible, it can be further developed into a more comprehensive tool covering in more detail legal issues. For a data provider who offers data as a service and thus takes on the role of a “data controller” the recommendations LAT produces for compliant data sharing can be the first step and must be completed by consideration of relevant local laws and regulations. Until now LAT considers only the EU framework and UK regulations. Such a complementation of LAT with national regulations is in agreement with the legal framework of RIs, for example with the principle of employing local regulations called “Home State Compliance” by BBMRI. Under this principle, each BBMRI member has to secure and guarantee that operations within the research infrastructure, especially the cross-border transfer of data and biosamples, are compliant with the member's own national laws [45].

As mentioned above, legal compliance of research with sensitive data is of growing concern for the research community. There exists a considerable need in the Life Sciences for easy to apply guidance for the secure and compliant handling of human health data. Many researchers as well as experts in data protection agree that the sharing of sensitive data needs to be planned from the start of a research project in order to be successful [46]. As shown in this paper, sharing research data comes with many ethical and legal issues. Since these issues are often complex, they can rarely be solved with one size fits all solutions [46]. In general, the complete anonymization of research data before any sharing process is in general being recommended; but often this is not possible. Especially for data, which cannot be anonymised, strict governance procedures as part of a data protection framework to restrict data access and usage, is needed. Many groups have developed some form of guidance to tackle these problems. To help to design and implement data governance structures including the ones for encryption, identity management, safe havens, data brokers, etc., a zone model consisting of three privacy protection zones (Care Zone, Non-Care Zone and Research Zone) provides a graphic reference system to help design privacy frameworks [47]. In addition, access may be based on explicit consent (with an option to agree or disagree with the collection, processing, or disclosure of personal information) or on country-specific or local regulations (e.g., exemptions to consent for research) usually allowing for an opt-out regime. In this context, funder organisations, universities and research infrastructures have developed guidelines how to deal with sharing of research data including sensitive data for research, for example University of California, ANDS (Australian National Data Service), UCL (University College London), BBMRI, ECRIN, Wellcome Trust [48–53]. For example, the Regulatory Affairs Database of ECRIN [53] provides advice to people who want to plan mono- or multi-centre clinical trials within different European countries including links to legal documents and the contact addresses of national authorities of 15 European countries. In addition, ELSI (Ethical, Legal and Social Issues) sources provide information about the requirements for using and sharing genetic human data. ELSI2.0 aims to encourage international collaboration and discussion around the Ethical, Legal and Social Implications of research in the Life Sciences [54]. HumGen is an international database on ethical, legal and social issues in human genetics including a database of laws and policies [55]; BioPolicy is a Wiki guide to laws and policies governing the use of human genetic and reproductive technologies [56]; ELSI Genetics Resource Directory (ELSI ReD) has been developed to provide a source to locate documents on genetic testing and screening, pharmacogenomics, genetic patents, genetic databanks and gene therapy with the focus on ethical and legal issues raised by the international transfer of data and results [57]. The U.S. Department of Energy (DOE) and National Institutes of Health (NIH) support a large bioethics program [58] and The Center for Transdisciplinary ELSI Research in Translational Genomics (CT2G) brings together different resources and serves as a repository for ethical, legal and social analysis of translational genomics [59].

For our analysis we focused on four resources that offer some kind of interactive user access to their documents: on P3G-IPAC (International Policy interoperability and data Access Clearinghouse) Generic Clauses Database [60], BBMRI Legal WIKI [61], Human Sample Exchange Regulation Navigator (hSERN) [62] and Treat-NMD (Translational Research in Europe for the Assessment and Treatment of Neuromuscular Disease) Regulatory Affairs Database [53].

The International Policy interoperability and data Access Clearinghouse (IPAC) [60] provides services for policy interoperability and access authorization. On the other hand, the BBMRI Legal WIKI [61] offers templates for European biobanking research, like standard personal data processing security agreements and material transfer policies. hSERN [62] provides information on theoretical and practical legal aspects for exchanging biosamples across borders. All these sources, more or less, provide the user with general legal information, links to the relevant acts and ordinances and may even provide templates for documents like data sharing agreements. At the first sight helpful, such resources may burden most researchers with the need to study and interpret legal documents. Thus, in our opinion, the mentioned lists of national regulations offer little help for the concerned researcher, because one cannot expect that a researcher for each data sharing problem consults a list of regulations and reads through all legal texts. In contrast, our approach focuses on data access and usage rules employed by various data providers and treats them as requirements for the data sharing processes. This approach is possible, because all data providers are subordinate to the corresponding national rules and regulations and responsible that at their database data protection for sensitive human data is enforced and consequently, they have already done their work and read and interpreted the relevant legal texts.

This request for simplicity was mirrored in the reception of the tool by the BioMedBridges community. The usability of the tool was evaluated by interviews and hands-on test sessions with potential users at several BioMedBridges project meetings. The researchers coming from diverse research areas, like mouse phenotypic research, cancer research, personalised medicine, commented on the tool during testing. These comments were used to improve the user interface. In summary, the tool was judged by researchers to be very useful concerning data protection and/or ethical issues and especially the support in the form of guidance in the case of uploading of potentially identifying data to an open access data source was appreciated [63]. In addition, during several telephone conferences with BioMedBridges members and experts, the usability of LAT was further improved, with the aim to make the use as easy as possible and to show results of the selection of criteria as immediate as possible. Sections for "How to use this tool" and "Frequently Asked Questions" were added, explaining each query question of the tool and commenting on specific issues, for example: "Is there a legal basis to share the data or biosamples without specific consent?" While researchers were largely positive, some even enthusiastic about the tool, for many legal experts and data security specialists the tool was too simplistic and the direct consideration of legal texts on European and national levels was missed. The positive feedback from researchers encouraged us to continue in the direction of creating a simple tool usable without legal knowledge, but also to think about extending the tool to include more local and national regulations, a further development that is much more demanding and will have to involve the contributions of many legal experts from different countries. To support this approach, BBMRI with its Common Service ELSI consisting of legal experts from its RI member countries offered to adopt the tool and to integrate it with other tools such as hSERN and the BBMRI Legal WIKI.

Nonetheless, LAT is a proof-of-concept application and still has some shortcomings. But before implementing further improvements, attention should be taken not to abandon the simplicity and easy usability that characterises LAT. Finally, it should be stressed that LAT is not intended to provide legal counselling, but acts on a level higher; a level where legal text is already interpreted and presented as rules and recommendations.

## Conclusions

It cannot be the responsibility of the researcher who wants to access data to handle the legal intricacies of EU and national data protection legislations; this must be done by the data provider who acts as a data controller and who is the only one, who can check, whether any intended data sharing is compliant with his local laws including ethics committees votes and patients’/donors’ consent. The data controller must respect a set of rules as set out, for example, in the Data Protection Directive [64], like that personal data is processed legally and fairly, the data is collected for explicit and legitimate purposes and used accordingly and the data collection is adequate, relevant and not excessive in relation to the purposes. To this effect, LAT builds on this obligation and assumes that the corresponding data controller has respected them. Nonetheless, BioMedBridge's data bridges should contribute to a simplification and harmonization of data sharing conditions and support open access as far as possible. Data bridges must be opened for human health data and LAT is one of the means to further this aim. Here we show that our pragmatic approach to build on the available data access and processing rules of established databases does work and facilitates considerably the search for compliant data sharing requirements. We could demonstrate that IT concepts can be successfully applied to the legal domain allowing the use of concepts like “requirement cluster” and “legal interface” as part of data sharing between different data providers and data consumers. For the researcher querying the tool the provided information is for the most part sufficient and complete. This is based on our pragmatic approach to consider the data sharing rules of all data providers in BioMedBridges and all possible combinations between them. Nonetheless, gaps are present, not all rules are linked to source legal documents, and neither national legal peculiarities (with the exception of United Kingdom, UK) nor different forms of informed consent and committee approvals are considered. In addition, a more specific questionnaire and the generation of more specific response information would improve its usefulness for researchers. Nonetheless, LAT is a first step to deal with legal requirements in an interactive way. It is one of its achievements that by providing requirements for compliant data access and sharing with appropriate safeguards, restrictions and responsibilities, it is introducing a culture of responsibility and data governance when dealing with human data, and even with non-human data.

## Abbreviations

AMIA, American medical informatics association; ANDS, Australian National data service; BBMRI, biobanking and Biomolecular resources research infrastructure; BioSD, biosamples database; CERM, Centro di Ricerca di Risonanze Magnetiche; EATRIS, European infrastructure for translational medicine; ECRIN, European Clinical Research Infrastructures Networks; EGA, European Genome Atlas; ELIXIR, distributed infrastructure for life-science information; ELSI, ethical, legal and social implications; EM, electron microscopy; EMDB, electron microscopy data bank; ESFRI, European strategy forum on research infrastructures; FIMM, finnish institute for molecular medicine; GCP, good clinical practice; GEO, gene expression omnibus; GEP, good epidemiological practice; GLP, good laboratory practice; GWAS, genome-wide association study; hSERN, human sample exchange regulation navigator; HumGen, database on ethical, legal and social issues in human genetics; ICGC, international cancer genome consortium; ICH, international conference on harmonisation; ICO, information commissioner’s office (UK); iDASH, integrating data for analysis, anonymization, and sharing; IDs, identifiers; IEA, International Epidemiological Association; IMIA, International Medical Informatics Association; IP, intellectual property; IPAC, International Policy Interoperability and Data Access Clearinghouse; KKS, koordinierungszentrum für klinische studien; LAT, legal assessment tool; LDSG, landesdatenschutzgesetz (Germany); MS, mass spectrometry; MVC, model-view controller; OA, open access; OECD, Organisation for Economic Co-operation and Development; OPD, object property dependency; OWL, Web ontology language; P3G, public population project in genomics and society; PDBe, protein data bank in Europe; PET, privacy enhancing technology; PS, privacy service; Q&A, questions and answers; R, restricted access; RI, research infrastructure; rqNR, requirement number; SWRL, semantic web rule language; TCGA, the cancer genome atlas; TMF, telematikplattform (TMF e.V., Berlin, Germany); Treat-NMD, translational research in Europe for the assessment and treatment of neuromuscular disease; UCL, University College London.

## Additional file

Additional file 1: Table S1-S5. Tables describing in detail databases and their access policies considered for the requirement clusters of data bridges. (DOC 79 kb)

## References

[1] Obligations of data controllers. http://ec.europa.eu/justice/data-protection/data-collection/obligations/index_en.htm. Accessed 27 Apr 2016.

[2] van Panhuis WG, Paul P, Emerson C, Grefenstette J, Wilder R, Herbst AJ (2014). Heymann D and Donald S Burke. A systematic review of barriers to data sharing in public health. BMC Public Health.

[3] Lee LM, Gostin LO (2009). Ethical collection, storage, and use of public health data: a proposal for a national privacy protection. JAMA.

[4] IMIA Code of Ethics for Health Information Professionals. http://www.imia-medinfo.org/new2/node/39. Accessed 27 Apr 2016.

[5] Goodman KW, Adams S, Berner ES, Embi PJ, Hsiung R (2013). AMIA’s code of professional and ethical conduct. JAMIA.

[6] Shabani M, Borry P (2015). Challenges of web-based personal genomic data sharing. Life Sciences, Society and Policy.

[7] Knoppers BM, Harris JR, Budin-Ljøsne I, Dove ES (2014). A human rights approach to an international code of conduct for genomic and clinical data sharing. Hum Genet.

[8] Malin B, Karp D, Scheuermann RH (2010). Technical and policy approaches to balancing patient privacy and data sharing in clinical and translational research. J Investig Med.

[9] Yu F, Ji Z (2014). Scalable privacy-preserving data sharing methodology for genome-wide association studies: an application to iDASH healthcare privacy protection challenge. BMC Med Inform Decis Mak.

[10] Jiang X, Zhao Y, Wang X, Malin B, Wang S, Ohno-Machado L, Tang H (2014). A community assessment of privacy preserving techniques for human genomes. BMC Med Inform Decis Mak.

[11] BioMedBridges. www.biomedbridges.eu/. Accessed 27 Apr 2016.

[12] ESFRI (European Strategy Forum on Research Infrastructures). http://ec.europa.eu/research/infrastructures/index_en.cfm?pg=esfri. Accessed 27 Apr 2016.

[13] Ohmann C, Kuhn K and WP5: Deliverable D5.1. Tool for the assessment of regulatory and ethical requirements. BioMedBridges (31 December 2013). http://www.biomedbridges.eu/sites/biomedbridges.eu/files/documents/deliverables/d5-2_report_biomedbridges_deliverable_assessment_tool_edited _final_complete.pdf. Accessed 27 Apr 2016.

[14] Information Management Glossary, SourceMedia (2016). http://www.information-management.com/glossary/d.html. Accessed 27 Apr 2016.

[15] Large human databases with human data have been created, like the Human Metabolome Database (HMDB), Immuno Polymorphism Database, 1000 Genomes Project, European Genome-phenome Archive. http://www.hmdb.ca/, https://www.ebi.ac.uk/ipd/. http://www.1000genomes.org/, https://www.ebi.ac.uk/ega/home. Accessed 27 Apr 2016.

[16] ESFRI (2013). European Research Infrastructures with global impact.

[17] e-IRG Report on Data Management. Data Management Task Force. Espoo, Finland: e-IRG secretariat; 2009.

[18] Uhlir PF. The Legal Interoperability of Data. NSGIC Conference, 24–27 Feb 2013, Annapolis, MD, USA (2013). http://www.nsgic.org/public_resources/02_Uhlir_Legal-Interoperability-of-Data_NSGIC-Conf_Feb13.pdf. Accessed 27 Apr 2016.

[19] SMART2007/0059. Study on the legal framework for interoperable eHealth in Europe. Final report. Version 1.5. Brussels: European Commission (2009).

[20] Bartling S, Friesike S (2014). Opening Science.

[21] Pohl K (1997). Requirements Engineering: An Overview. Encyclopedia of Computer Science and Technology.

[22] Sutcliffe A. Scenario-based requirements engineering. Requirements Engineering Conference 2003. Proceedings. 11th IEEE International; 2003. 320–329.

[23] Ian A, Neil M (2004). Scenarios, Stories, Use Cases. Through the systems development life-cycle.

[24] Interface (computing). https://en.wikipedia.org/wiki/Interface_(computing). Accessed 29 Apr 2016.

[25] Directive 95/46/EC of the European Parliament and of the Council of 24 October 1995. No L 281/31. Luxembourg, Luxembourg; 1995.

[26] Guideline for Good Clinical Practice E6(R1). ICH Expert Working Group; 1996.

[27] World Intellectual Property Organisation (WIPO). http://www.wipo.int/ipstrategies/en/. Accessed 26 Apr 2016.

[28] Boussi Rahmouni H, Solomonides T, Casassa Mont M, Shiu S, Rahmouni M (2011). A model-driven privacy compliance decision support for medical data sharing in Europe. Methods Inf Med.

[29] Ramingwong L (2012). A review of requirements engineering processes, problems and models. Int J Eng Sci Technol (IJEST).

[30] Legal Assessement Tool (LAT). http://hhu2.at.xencon.de:8080/lat/tool. Accessed 26 Apr 2016.

[31] Train-online LAT. http://www.ebi.ac.uk/training/online/. Accessed 26 Apr 2016.

[32] The Ethical Governance Framework of BioMedBridges. http://www.biomedbridges.eu/deliverables/16. Accessed 26 Apr 2016.

[33] de Maat E, van Engers TM. Mission impossible? Automated norm analysis of legal texts. Legal Knowledge and Information systems, Jurix. 2003 (sixteenth Annual Conference); 2003. p.398.

[34] Gaur S, Vo NH, Kashihara K, Baral C. Translating Simple Legal Text to Formal Representations (2015). http://www.public.asu.edu/~cbaral/papers/shruti2015.pdf. Accessed 26 Apr 2016.

[35] Poulin D, Bratley P, Frémont J, Mackaay E. Legal interpretation in expert systems. In: Proceedings of the 4th international conference on Artificial intelligence and law. ACM; 1993. pp. 90–99.

[36] Grabmair M, Ashley KD. Towards Modeling Systematic Interpretation of Codified Law. In: Moens MF, Spyns P, editors. Legal Knowledge and Information Systems (JURIX 2005). Amsterdam: IOS Press; 2005. p. 107-8.

[37] Breaux TD, Vail MW, Antón A. Towards regulatory compliance: Extracting rights and obligations to align requirements with regulations. In: Requirements Engineering, 14th IEEE International Conference Proceedings (IEEE 2006, September); 2006:49–58.

[38] Casellas N, Nieto JE, Meroño A (2010). Ontological Semantics for Data Privacy Compliance: The NEURONA Project.

[39] Cappelli A, Lenzi VB, Sprugnoli R, Biagioli C. Modelization of Domain Concepts Extracted from the Italian Privacy Legislation. In: Proceedings of the Workshop on Computational Semantics (IWCS-7); 2007. http://www.ittig.cnr.it/Presentazione/OrganizzazioneLogistica/biagioli/Cappelli-et-al.pdf. Accessed 26 Apr 2016.

[40] LKIF-Core Ontology - core ontology of basic legal concepts. http://www.estrellaproject.org/lkif-core/. Accessed 26 Apr 2016.

[41] Allison DS, Capretz MAM, ELYamany HF, Wang S (2012). Privacy protection framework with defined policies for service-oriented architecture. J Softw Eng Appl.

[42] McCallister E, Grance T, Scarfone K (2010). Guide to Protecting the Confidentiality of Personally Identifiable Information (PII). NIST, Special Publication 800–122.

[43] Rahmouni HB, Solomonides T, Mont MC, Shiu S (2009). Privacy compliance in European healthgrid domains: an ontology-based approach. Proc. 22nd IEEE Int. Symp.

[44] Rahmouni HB, Solomonides T, Mont MC, Shiu S. Ontology-based privacy compliance on European healthgrid domains. In: Proc. 11th Int. Protégé Conf. Amsterdam, The Netherlands, 23–26 June 2009; 2009. http://protege.stanford.edu/conference/2009/abstracts/S13P1Boussi.pdf for extended abstract. Accessed 26 Apr 2016. 19593056

[45] Home State Compliance. http://www.bbmri-wp4.eu/wiki/index.php/Home_State_Compliance. Accessed 26 Apr 2016.

[46] Nielsen F, Teperek M. How to share personal/sensitive research data? Repositive, Blog, 29 February 2016, University of Cambridge. Cambridge UK: Future Business Centre. http://blog.repositive.io/how-to-share-personal-sensitive-research-data/. Accessed 26 Apr 2016

[47] Kuchinke W, Ohmann C, Verheij RA, van Veen EB, Arvanitis TN, Taweel A, Delaney BC (2014). A standardised graphic method for describing data privacy frameworks in primary care research using a flexible zone model. Int J Med Inform.

[48] Expert Advisory Group on Data Access: Governance of Data Access. London UK: Wellcome Trust (June 2015). http://eprints.whiterose.ac.uk/92286/1/wtp059343.pdf. Accessed 26 Apr 2016.

[49] Governance of data access**,** London UK: Wellcome Trust. http://www.wellcome.ac.uk/About-us/Policy/Spotlight-issues/Data-sharing/EAGDA/wtp059350.htm. Accessed 26 Apr 2016.

[50] Data Best Practices. Research data. Working with Sensitive Data. Berkeley, CA, USA: University of California Berkeley. http://researchdata.berkeley.edu/content/working-sensitive-data. Accessed 26 Apr 2016.

[51] ANDS (Australian National Data Service) Guide: Ethics, consent and data sharing. http://ands.org.au/guides/ethics-consent-and-data-sharing. Accessed 25 Apr 2016.

[52] Bhimani N (2016). Personal and sensitive research data & the law. UCL Blog (22 January 2016).

[53] Regulatory Affairs Database. TREAT-NMD. http://www.treat-nmd.eu/industry/regulatory-affairs/. Accessed 28 Apr 2016.

[54] ELSI2.0 workspace. https://elsi2workspace.tghn.org/. Accessed 28 Apr 2016.

[55] HumGen: International Database on the Legal, Social, and Ethical Aspects of Human Genetics. http://www.humgen.org/. Accessed 01 June 2016.

[56] BioPolicy Wiki. http://www.biopolicywiki.org/index.php?title=Main_Page. Accessed 01 June 2016.

[57] WHO's ELSI Genetics Resource Directory. http://www.who.int/genomics/elsi/regulatory_data/en/. Accessed 28 Apr 2016.

[58] US. DOE ELSI Research. http://www.ornl.gov/sci/techresources/Human_Genome/research/elsi.shtml. Accessed 25 Apr 2016.

[59] Center for Transdisciplinary ELSI Research in Translational Genomics (CT2G). http://www.ct2g.org/resources.html. Accessed 28 Apr 2016.

[60] The International Policy interoperability and data Access Clearinghouse (IPAC) provides a “one stop” screening service for policy interoperability and access authorization. http://www.p3g.org/ipac. Accessed 28 Apr 2016.

[61] BBMRI’s legal wiki. http://www.bbmri-wp4.eu/wiki/index.php/Main_Page. Accessed 25 Apr 2016.

[62] hSERN (Human Sample Exchange Regulation Navigator). http://bbmri-eric.eu/events/-/asset_publisher/wiZaUl5ie56w/content/webinar-hsercn. Accessed 29 Apr 2016.

[63] Tool for assessment of regulatory and ethical requirements. BioMedBridges (2015). http://www.biomedbridges.eu/sites/biomedbridges.eu/files/documents/deliverables/user-guide_and_tool-description_biomedbridges_legal-assessment-tool.pdf. Accessed 25 Apr 2016.

[64] EU Data Protection Collection. http://ec.europa.eu/justice/data-protection/data-collection/obligations/index_en.htm. Accessed 25 Apr 2016.

[65] Brittain J, Darwin IF (2007). Tomcat: The Definitive Guide.

[66] Lindholm T, Yellin F (1999). Java Virtual Machine Specification.

