# Supplementary material for: Legal assessment tool (LAT): an interactive tool to address privacy and data protection issues for data sharing
Source: BMC Med Inform Decis Mak. 2016 Jul 7;16:81. doi: 10.1186/s12911-016-0325-0 (PMC5067915; doi:10.1186/s12911-016-0325-0)
Supplement: Additional file 1: Table S1-S5. — Tables describing in detail databases and their access policies considered for the requirement clusters of data bridges. (DOC 79 kb) [file 12911_2016_325_MOESM1_ESM.doc]

**Supplementary Tables**

**Tables 1-5: Databases and their access policies considered for the requirement clusters of data bridges**

Wolfgang Kuchinke, Christian Krauth, René Bergmann, Töresin Karakoyun, Astrid Woollard, Irene Schluender, Benjamin Braasch, Martin Eckert, Christian Ohmann. Legal Assessment Tool (LAT): An interactive tool to address privacy and data protection issues for data sharing.

**Table 1**

| Database | | | Access policy |
| --- | --- | --- | --- |
| Data providers | | | |
| **Mouse tissue imaging data (Infrafrontier)** | | Restricted access  Data linking only possible, if the data provider gives permission based on the availability of informed consents given by research participants  Every data provider has to be asked if informed consent exist  **Personal data of researcher/ data collector is excluded** | |
| **Human tumour tissue data (BBMRI/ FIMM)** | | Restricted access  Accessible through an application to the steering committee or principal investigator of the respective project^1^  A consent form which permits such a research is required if informed consent does not cover the envisioned type of research  **Images are owned by image generator, they must approve using the data** | |
| **MitoCheck (cell-based RNAi screens):** | | Open access, no usage limitations | |
| **WebMicroscope.net (mouse and human image data sets):** | | Restricted access, authentication necessary, login only possible for BMB members  **Access to WebMicroscope**^2^  **will be protected with rights management; therefore, different access tiers can be established** | |
| **Ensembl** | | Open access, no usage limitations to a specific purpose | |
| **ArrayExpress** | | Open access, no usage limitations to a specific purpose | |
| Data consumers | | | |
| WebMicroscope | Restricted access | | |
| Phenotator | Open/ open restricted  Annotations can be done private or public by anyone. Private annotations can be accessed with username/ password  For this usage scenario the annotations are public. | | |
| **MitoCheck:** | Open access | | |
| https://www.fimm.fi/en/services/biobanking-infrastructure/mibi/mibi-policies-and-costs  ^2^ Web-based Virtual Microscopy: a method of digitizing microscope specimens, and viewing the produced virtual slides on a computer screen. | | | |

**Table 2**

| Database | Access policy |
| --- | --- |
| Data providers, Data consumers | |
| **EuroPhenome** | **Open access** |
| **IMPC** | **Open restricted** |
| **Gene Expression Atlas (GXA)/ ArrayExpress** | Open access, open restricted for private data (pre-publication/ unpublished) |
| **ChEMBL** | **Open access** |
| **Metabolights** | **Open access** |
| **Reactome** | Open access, data can be used for research (used for mapping in data bridges) |
| CERM datasets | Restricted access |
| **Biobank/ BBMRI (University of Graz)** | Restricted access, defined access rules including project applications and approval committee, mouse data, Human data |

**Table 3**

| Database | | Access policy |
| --- | --- | --- |
| Data providers | | |
| **ICGC (International Cancer Genome Consortium)[[1]](#footnote-2)** | Data with and without restricted access, different policies apply, both will be used for the usage scenario  Open access datasets: open access  Controlled access datasets: restricted access  Data Access Compliance Office (DACO) handles requests from scientists for access to controlled data from the ICGC  Access control is explained on ICGC website[[2]](#footnote-3):   - Data Access Compliance Office (DACO) handles requests from scientists for access to controlled data - user authentication via access request - Informed consent: can be used in cancer research, may include whole genome sequencing - potentially identifying data (defined by ICGC) will only be used by qualified scientists for public health objectives - Authorizations by the DACO to access controlled data is broad, so that authenticated users will get permission to obtain access to controlled data generated from all samples studied by any participating cancer genome project Redistribution is not allowed   For samples stored at ICGC the required consent should have been obtained if necessary. ICGC gives guidelines for the appropriate consent for prospective research, and for retrospective research, which should be considered by ICGC members.[[3]](#footnote-4) | |
| TCGA (The Cancer Genome Atlas)[[4]](#footnote-5) | Data with and without restricted access, both will be used in the usage scenario  Open Access, data publicly available  Controlled Access Data Tier: Data with restricted access, access control as explained on their website[[5]](#footnote-6)  Requires user certification via Data Access Request  Restricted to biomedical research purposes only  Researchers and their institution must agree TCGA Data User Certification (DUC) (includes not to distribute data): TCGA Approved User | |
| **EGA (European Genome-phenome Archive)[[6]](#footnote-7)** | Restricted access  Download of datasets must be approved by the specified Data Access Committee (DAC)  Requires users to sign a Data Access Agreement (DAA), which details the terms and conditions of use for each dataset  All controlled access downloadable datasets are encrypted using Bcrypt (.bfe files) or GnuPG (.gpg files) | |
| **Cosmic (Catalogue of somatic mutations in cancer)[[7]](#footnote-8)** | Open access  Wellcome Trust Sanger Institute data sharing policy[[8]](#footnote-9): Data sharing policies will only be sustainable, if researchers commit to:   - Conducting appropriate research - Respecting rights to first publication and acknowledgement - Sharing the resulting data and analysis with the research community | |
| **GEO (Gene Expression Omnibus)[[9]](#footnote-10)** | Open access, no usage limitations to a specific purpose | |
| **Array-Express[[10]](#footnote-11)** | Open/ restricted access, no usage restrictions, weekly imports of GEO data  Restricted access usually for pre-published/ unpublished data  Only open access data are used in this usage scenario | |
| **ChEMBL[[11]](#footnote-12)** | Open access | |
| **Reactome[[12]](#footnote-13)** | Open access | |
| **Ensembl[[13]](#footnote-14)** | Open access | |
| **Drugbank[[14]](#footnote-15)** | Open access. Use and re-distribution of the data for commercial purposes requires explicit permission of the authors and explicit acknowledgment of the source material. | |
| **Pharmgkb[[15]](#footnote-16)** | Open access, freely available to researchers in academia and industry for research purposes  Only for educational and scientific research purposes  Not available for redistribution  If it is planned using any contents from the PharmGKB for a product or with a plan to redistribute it (e.g., via a website), then a separate license must be obtained  Contains anonymised data | |
| **BioSD[[16]](#footnote-17)** | Open access  Open access data contains links to appropriate databases, like ArrayExpress  For anonymised, aggregated sample information contact information is provided, to be able to get access to more data | |
| Biobanks (BBMRI) | Restricted access, depending on biobank | |
| EU-OPENSCREEN | Open access | |
| ECRIN (CTIM) | Open access | |
| FIMM^17^ | Open (anonymous data) / restricted access (personal data); provides a service to query FIMM data  Additionally a PM data analysis informatics pipeline will be built. Different access tiers will be part of it | |
| Data consumers | | |
| FIMM (EATRIS) | Data consumer  Has built a database with restricted access at FIMM institute (Institute for Molecular Medicine Finland) for AML research.  They already have personal and clinical data of AML patients, which will be enriched by the newly linked data.  The ELIXIR (open access) databases BioSD and ChEMBL are data consumer. | |
| 1 http://icgc.org/  2 http://icgc.org/icgc/goals-structure-policies-guidelines/e1-informed-consent-access-and-ethical-oversight  3 http://www.icgc.org/icgc/goals-structure-policies-guidelines/e1-informed-consent-access-and-ethical-oversight  4 https://tcga-data.nci.nih.gov/tcga/  5 https://tcga-data.nci.nih.gov/tcga/tcgaAccessTiers.jsp  6 https://www.ebi.ac.uk/ega/  7 http://cancer.sanger.ac.uk/cancergenome/projects/cosmic/  8 http://www.sanger.ac.uk/datasharing/  9 http://www.ncbi.nlm.nih.gov/geo/  10 http://www.ebi.ac.uk/arrayexpress/  11 https://www.ebi.ac.uk/chembl/  12 http://www.reactome.org/ReactomeGWT/entrypoint.html  13 http://www.ensembl.org/index.html  14 http://www.drugbank.ca/  15 http://www.pharmgkb.org/  16 http://www.ebi.ac.uk/biosamples/index.html  17 https://www.fimm.fi/ | | |

**Table 4**

| Database | Access policy |
| --- | --- |
| Data providers | |
| UniProt**[[17]](#footnote-18)** | Open access |
| AmiGO**[[18]](#footnote-19)** | Open access |
| EMDB**[[19]](#footnote-20)** | Open access |
| IntAct**[[20]](#footnote-21)** | Open access |
| GenBank (NCBI)**[[21]](#footnote-22)** | Open access |
| Data consumers | |
| ELIXIR | EMDB: open access |
| BMB database | Planned as open access |
| ^17^ http://www.uniprot.org/  ^18^ http://amigo.geneontology.org/cgi-bin/amigo/go.cgi  ^19^ http://www.ebi.ac.uk/pdbe/emdb/  ^20^ http://www.ebi.ac.uk/intact/  ^21^ http://www.ncbi.nlm.nih.gov/genbank/ | |

**Table 5**

| Database | Access policy |
| --- | --- |
| Data providers | |
| **Nordic biobanks[[22]](#footnote-23)**  **French BIOBANQUES consortium (BBMRI) BBMRI Finland biobank** | Restricted access (via EGA for Nordic biobanks) |
| **BioSD[[23]](#footnote-24) (ELIXIR)** | Open access  Shows only aggregated anonymised information or metadata about sample collections or individual samples  Contact information provided |
| Data consumers | |
| BioSD | Open access |
| Researcher | Has to apply for data access to Data Access Committee (DAC) of biobank  Has to agree, using data only for contracted research, which has been specified between biobank and researcher, and not to try to disclose patient identity etc. |
| ^22^http://www.nordicdb.org/database/Home.html  ^23^http://www.ebi.ac.uk/biosamples/index.html | |

1. [↑](#footnote-ref-2)
2. [↑](#footnote-ref-3)
3. [↑](#footnote-ref-4)
4. [↑](#footnote-ref-5)
5. [↑](#footnote-ref-6)
6. [↑](#footnote-ref-7)
7. [↑](#footnote-ref-8)
8. [↑](#footnote-ref-9)
9. [↑](#footnote-ref-10)
10. [↑](#footnote-ref-11)
11. [↑](#footnote-ref-12)
12. [↑](#footnote-ref-13)
13. [↑](#footnote-ref-14)
14. [↑](#footnote-ref-15)
15. [↑](#footnote-ref-16)
16. [↑](#footnote-ref-17)
17. [↑](#footnote-ref-18)
18. [↑](#footnote-ref-19)
19. [↑](#footnote-ref-20)
20. [↑](#footnote-ref-21)
21. [↑](#footnote-ref-22)
22. [↑](#footnote-ref-23)
23. [↑](#footnote-ref-24)
